# Retirement of Dr. Hazel Jones as Co-Editor-in-Chief of *Fluids and Barriers of the CNS.* Dr. Mark Hamilton becomes Co-Editor-in-Chief

**DOI:** 10.1186/s12987-023-00444-1

**Published:** 2023-05-29

**Authors:** Richard F. Keep, Lester R. Drewes

**Affiliations:** 1 Department of Neurosurgery, University of Michigan, R5018 BSRB 109 Zina Pitcher Place, Ann Arbor, MI 48109-2200 USA; 2 Department of Biomedical Sciences, University of Minnesota Medical School Duluth, Duluth, MN 55812 USA

Dr. Hazel Jones is retiring as Co-Editor-in-Chief of *Fluids and Barriers of the CNS* (as of May 1st, 2023). We are happy to announce that Dr. Mark Hamilton (University of Calgary) will become Co-Editor-in-Chief (joining Drs. Richard Keep and Lester Drewes). Dr. Hamilton, a neurosurgeon, is an internationally recognized expert on hydrocephalus and Past President of the Hydrocephalus Society (ISHCSF). He will have a particular role in overseeing submissions related to hydrocephalus and CNS fluids, as well as guiding the future of the journal. Dr. Hamilton is warmly welcomed as he assumes his new role.

**Fig. 1:**
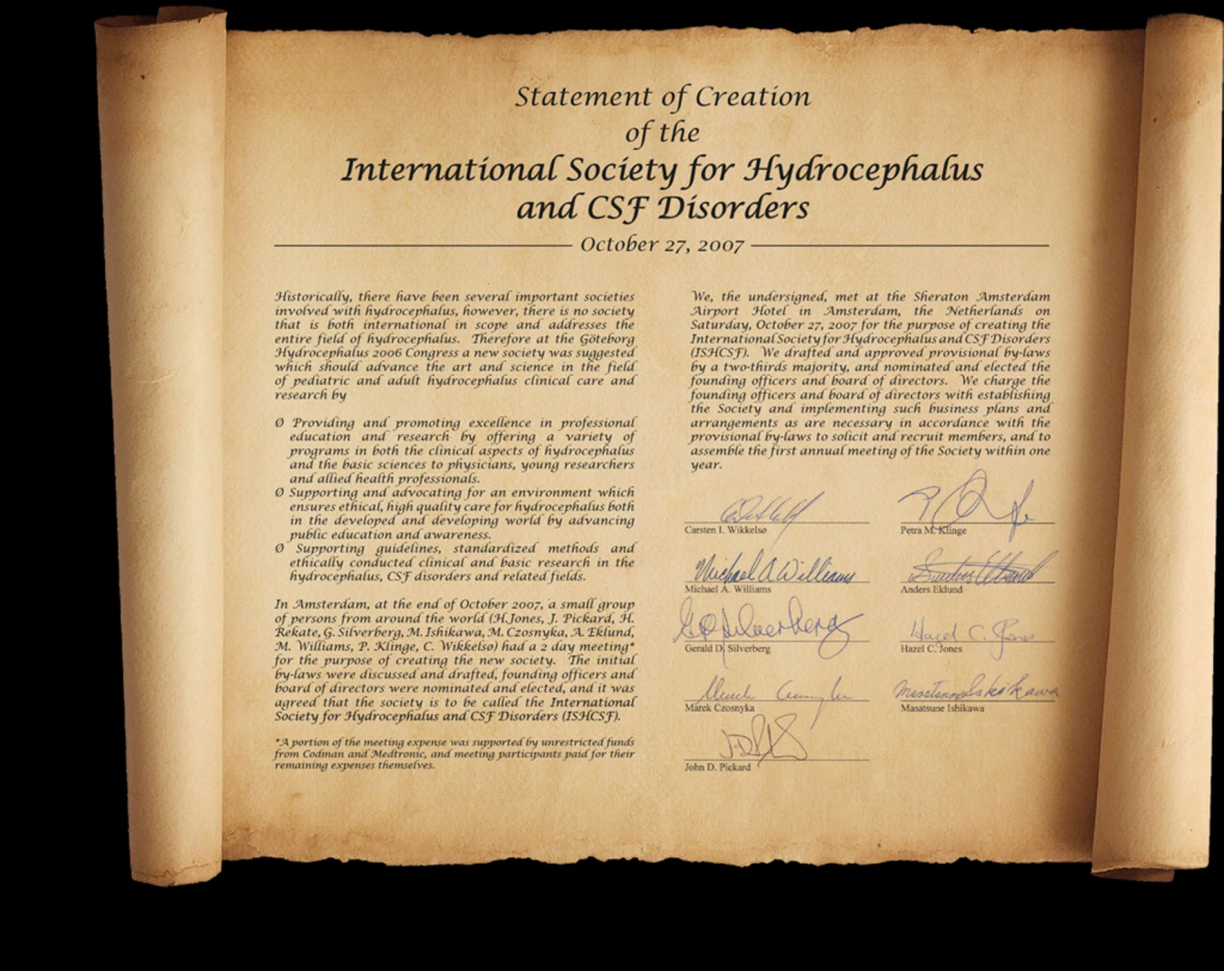
The founding statement for the International Society for Hydrocephalus and CSF Disorders (ISHCSF)

**Fig. 2:**
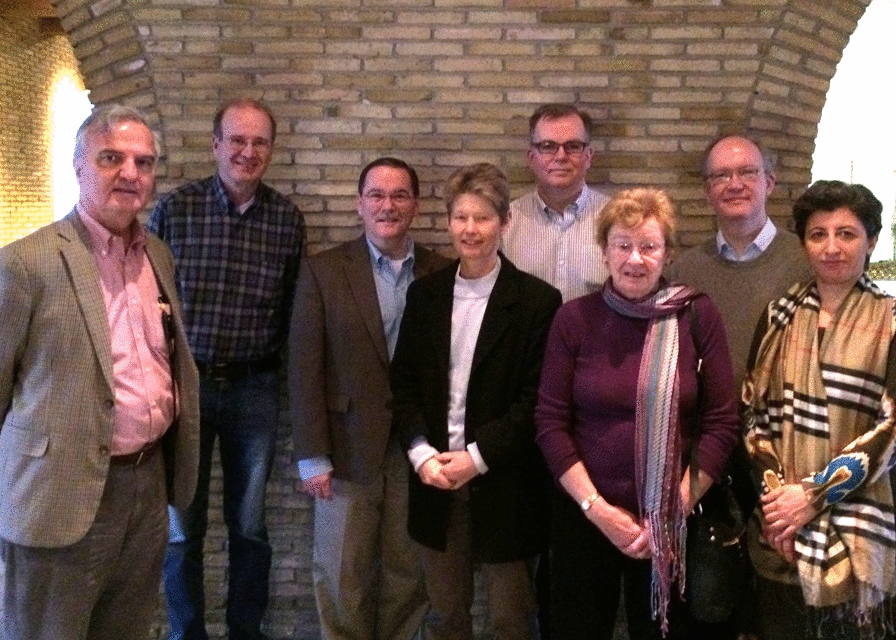
The International Society for Hydrocephalus and CSF Disorders (ISHCSF) Board at Copenhagen. From left, Daniele Rigamonti, Uwe Kehler, Mike Williams, Marianne Juhler, Mark Hamilton, Hazel Jones, Laurence Watkins, Gunes Aygok

Hazel was founding editor of what was then *CSF Research* in 2004 [1]. When she founded the open access journal at BioMed Central, she had recently retired from a career in CSF and experimental hydrocephalus research at the University of Hull, King’s College, London, and the University of Florida. The journal was originally designed as a vehicle for those wanting to publish CSF and hydrocephalus research and was first instigated by neurosurgeon Ian People and the Society for Research into Hydrocephalus and Spina Bifida under the presidency of neurosurgeon Carys Banister. In 2007, Hazel became involved with the founding and running of a new society: the International Society for Hydrocephalus and CSF Disorders (ISHCSF), now known as the Hydrocephalus Society (Figs. 1 and 2). In 2011, after consultation, Hazel decided to increase the scope of the journal by including a new focus on brain barriers research (blood-brain and blood-CSF barriers) and the title was changed to *Fluids and Barriers of the CNS*. At this stage, Richard Keep and Lester Drewes with their expertise in brain barriers research joined Hazel as Co-Editors-in-Chief. In 2015, the journal became officially affiliated to both the Hydrocephalus Society and the International Brain Barriers Society (IBBS).

The growth and success of the journal is fundamentally due to Hazel’s indomitable will and the endless hours she was willing to put in to foster its success. Starting a new journal is not easy! However, under Hazel’s guidance, the journal has grown from strength to strength. For example, in 2012, the journal was cited 243 times, in 2017 that was 1024 times, and in 2022 it was cited 3002 times, a 12-fold increase over a decade. There were also about 520,000 downloads in 2021.

The journal was sorely needed. Many were finding it difficult to find a ‘home’ for their CSF/hydrocephalus/brain barrier research in ‘mainstream’ journals. Under Hazel’s guidance, the journal not only provided a home for such research, it also helped foster talent entering the field by providing awards for junior investigators attending hydrocephalus and brain barrier conferences. The current editors are ever grateful for Hazel Jones’ drive, determination and commitment to the highest standards of research publication. The whole field owes a great debt of gratitude to Hazel for her founding the journal and her leadership over nearly 20 years. She will be keeping an eye on the journal’s progress!
